# Sleeve Gastrectomy in a Severe Hemophilia A Patient: One of the Very Rare Cases

**DOI:** 10.4274/tjh.galenos.2020.2020.0168

**Published:** 2020-11-19

**Authors:** Eren Arslan Davulcu, Zühal Demirci, Özgür Fırat, Güray Saydam, Fahri Şahin

**Affiliations:** 1 Ege University Medical Faculty, Hematology Department, Ege Adult Hemophilia and Thrombosis Center, İzmir, Turkey; 2 Ege University Medical Faculty, Department of General Surgery, İzmir, Turkey

**Keywords:** Hemophilia A, Obesity, Bariatric surgery

## To the Editor,

Obesity is becoming a problem for aging hemophilia patients. The estimated prevalence of overweight and obesity in European and North American hemophilia patients is 31% [1]. Similarly to non-hemophiliacs, excessive weight has an adverse effect on the cardiovascular system and the psychological and musculoskeletal health of hemophilia patients. Bariatric surgery is advised to be considered for patients from the general population with a body mass index (BMI) of ≥40 kg/m^2 ^or BMI of ≥35 kg/m^2^ with comorbidities [2]. Although it is not contraindicated, there are limited data on bariatric surgery among hemophilia patients. There are only two hemophilia A patients reported in the literature who had bariatric surgery. The first one underwent a successful sleeve gastrectomy [3] and the other had mini-gastric bypass surgery [4].

A 55-year-old severe hemophilia A patient with arterial hypertension, diabetes mellitus, hyperlipidemia, obstructive sleep apnea, and morbid obesity (BMI of 42.45 kg/m^2^) decided to have obesity surgery because of inconclusive efforts at losing weight and obesity-related comorbidities. He had been receiving factor VIII prophylaxis at 6000 units/week for the last few years because conventional treatment at 4500 units/week was not sufficient for his frequent joint bleedings.

According to the Hemophilia Diagnosis and Treatment Guidelines of the Turkish Society of Hematology [5], the perioperative factor VIII target was calculated as 100%. Three days before surgery, his factor VIII level was 2.3% and factor VIII inhibitor was negative. He received factor VIII at 42 units/kg preoperatively and we planned to administer 23 units/kg at the postoperative 12^th^ hour (weight: 130 kg). At the postoperative 7^th^ hour he had hypotension, loss of consciousness, oliguria, 13% decline in hematocrit level, and increase in creatinine and transaminase levels. At that time, activated partial thromboplastin time (aPTT) was 26.8 s (normal range: 22.5-31.3). During explorative laparotomy, approximately 500 mL of blood was drained from the abdominal cavity, but a surgical bleeding focus could not be found. Packing was performed according to damage control surgery principles. After 48 h the abdominal cavity was reopened for unpacking and no bleeding was observed. He was followed in the intensive care unit for the following 8 days with mechanical ventilation. He needed intermittent hemodialysis because of hemorrhage-related acute kidney injury. During this period, he had thrombocytopenia and prolonged prothrombin time. He had multiple erythrocyte, platelet, and fresh frozen plasma replacements due to probable disseminated intravascular coagulation. Hemorrhage from the surgical drains lessened and finally stopped. Perioperative laboratory results are summarized in Table 1.

Factor VIII replacement was performed at 100% level (42 units/kg/day) until bleeding ceased for 8 days after surgery. The factor threshold was decreased to 60% (30 units/kg/day) during the next 8 days and to 40% (20 units/kg/day) the next 5 days, and then the patient was switched to prophylactic treatment (6000 units/week).

During follow-up in the inpatient clinic for the next 30 days, his need for high doses of factor VIII replacement diminished, his urine output increased, cytopenia and hemostatic problems resolved, and kidney and liver functions normalized.

At 7 months after surgery, he had lost 42 kg and his BMI was 28.7 kg/m^2^. His blood pressure and glucose are now well controlled. We intend to reduce factor VIII prophylaxis to 4500 units/week when the ideal body mass is achieved.

To the best of our knowledge, we are reporting the second hemophilia A patient to have a sleeve gastrectomy operation. The course of the procedure was challenging but the patient survived without any permanent damage and has even already begun to see the benefits of losing weight. His unexpected bleeding a couple of hours after the operation could not be attributed to any manifest surgical reasons or hemostatic defects. However, we think that there were likely some microvascular and hemostatic deficiencies. Since it was not possible to measure the factor VIII level just before the operation, we did not know the factor level precisely. Although aPTT was in the normal range, the factor concentration may be below the target level during this procedure. Our experience shows that major surgical operations such as bariatric surgery can be complicated in hemophilia patients; therefore, it is necessary that all parts of the team providing hemophilia care cooperate together.

## Figures and Tables

**Table 1:**
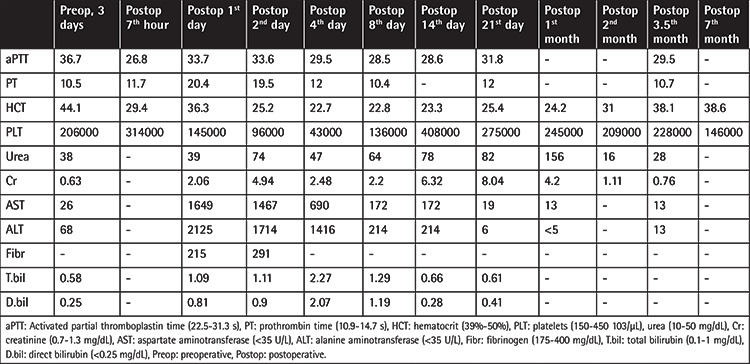
Perioperative laboratory results.
